# Electro-Superplastic Solid State Welding of 40Cr/QCr0.5

**DOI:** 10.3390/ma11071153

**Published:** 2018-07-06

**Authors:** Yaoli Wang, Guangxin Wang, Keke Zhang

**Affiliations:** 1 School of Materials Science and Engineering, Henan University of Science & Technology, Luoyang 471003, China; zhkeke@haust.edu.cn; 2 Research Center for High Purity Materials, Henan University of Science & Technology, Luoyang 471003, China; 3 Collaborative Innovation Center of Nonferrous Metals, Luoyang 471023, China

**Keywords:** copper alloys, steel, electro-superplastic solid-state welding, strength, metallurgical bonding

## Abstract

Hot-squeezed chrome bronze QCr0.5 and ultra-fine treated 40Cr steel have been successfully welded using an electro-superplastic solid-state welding technique. Results have shown that the tensile strength of a 40Cr/QCr0.5 weld joint can be greatly increased, up to or exceeding that of QCr0.5 base metal. The weld interface between 40Cr and QCr0.5 has achieved metallurgical bonding and there are less micro-gaps, thicker transition regions and more copper convexes and dimples on the fracture surface of the 40Cr side when applying an external electrical field of *E* = 3 kV/cm, as well as with other welding parameters, including no vacuum, no shield gas, a pre-pressure of 56.6 MPa, an initial strain rate of 1.5 × 10^−4^ s^−1^, a pressure welding temperature of 710–800 °C, and a pressure welding time of 0–8 min.

## 1. Introduction

Pure copper and copper alloys have a number of superior properties, such as excellent electrical conductivity, thermal conductivity, and forming property. When they are used with relatively cheaper steel to form so-called composite structure components, complementary advantages in both economy and performance can be obtained. However, it is very difficult for copper and steel to bond well by using the conventional fusion welding and brazing method, because there exists a rather large difference between these two materials in terms of their melting point, the coefficient of thermal conductivity, and the coefficient of linear expansion. A more promising welding technology which can solve this problem is the isothermal superplastic solid-state welding method, which does not have a vacuum or shield gas. This method has several advantages, such as its simple process, low cost, and high quality, which makes for solid bonding between the heterogeneous and/or dissimilar materials [1,2,3,4,5]. An external electrical field is known to be able to significantly influence the superplastic deformation and the distribution of vacancy concentration of different materials [6,7,8,9,10,11,12]. Some studies have shown that an electrical field can reduce the flow stress and strain-hardening rate (or accelerate strain-softening), as well as significantly suppressing the movement of cavitation during superplastic deformation [13,14,15,16,17,18]. Meanwhile, Cao et al. [19] found that the electric field could increase the room temperature tensile strength and elongation, and there may also be a prevention of the growth of crystal grains as well as reduction of the volume fraction of voids on the grain boundary by adopting the electric field during the superplastic deformation of 7475 aluminum alloy [19]. Therefore, a technique named electro-superplastic solid-state welding has been developed to conduct isothermal superplastic solid-state welding within an electrical field. This technique was successfully used to weld steel with steel. As described by Zhang et al. [20], an ultra-high carbon steel part with 1.6% C was welded to a 40Cr steel part by using electro-superplastic solid-state welding, which had an electrical field intensity of +3 kV/cm, temperature of 760–800 °C, prepressed stress of 56.6 MPa, initial strain rate of 1.5 × 10^−4^ s^−1^ and preheating time of 20 min. As reported by Zhang et al. [20], the joint strength was increased by 26.8%, compared to that without an electrical field which had a temperature of 780 °C. As for welding copper alloys with steel using the electro-superplastic solid-state welding technique, no reports have been found by a literature search. Therefore, this paper is believed to be the first to test the electro-superplastic solid-state welding technique for copper alloys with steel. Our focus is on the effect of the electrical field intensity and processing parameters on tensile strength, as well as the deformation strain of weld joints. The results obtained in this paper may provide an important test reference to new electro-superplastic solid-state welding procedures for copper alloys with steel in the future.

## 2. Experimental Materials and Procedures

### 2.1. Experimental Materials and Welding Samples

For this study, hot-squeezed chrome bronze QCr0.5 and hot-rolled and annealed 40Cr steel were used as experimental materials. Table 1 outlines the chemical compositions of the two materials. For the welding tests, cylindrical samples of the dimensions Φ15 × 25 mm were machined for both materials. After sanding with different sizes of metallographic sandpaper, the welding surfaces of the specimens were polished on a polishing machine for about 20 min. The roughness (Ra) of the weld surfaces was measured to be in the range of 0.103–0.187 µm. It should be noted that the weld surface of the QCr0.5 and 40Cr should be cleaned with alcohol and acetone before welding.

### 2.2. Electro-Superplastic Solid State Welding Tests

**Figure 1:**
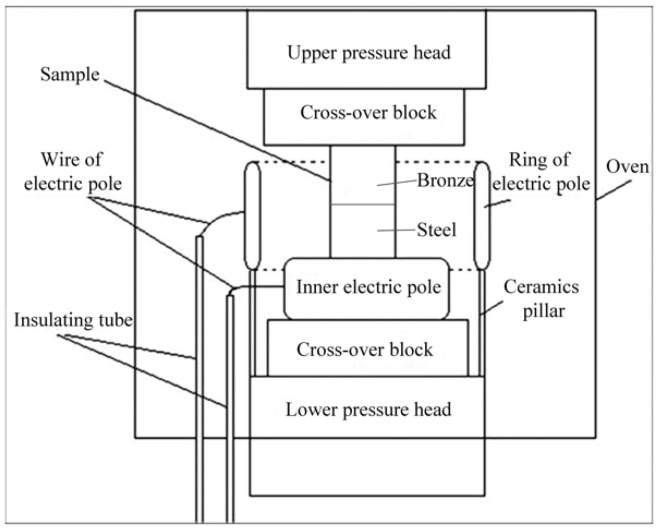
Schematic showing the setup of electro-superplastic solid state welding.

**Figure 2:**
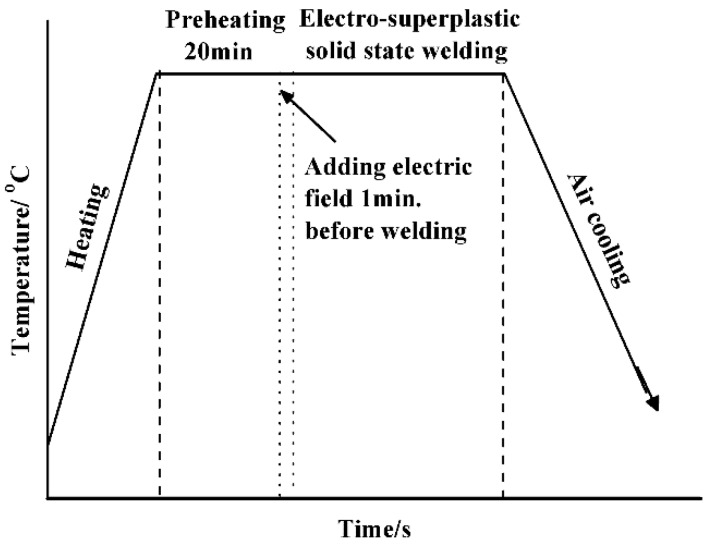
The process of electro-superplastic solid state welding.

Figure 1 is a schematic showing the experimental setup for electro-superplastic solid state welding. For each test, one QCr0.5 sample and one 40Cr sample were paired, packed and put into a pressure welding device. The samples were heated by a 3-kW electrical oven with a ±2 °C error of temperature and prepressed under a load of 1000 kg (i.e., a stress of 56.6 MPa for our cylindrical sample of the dimensions Φ15 × 25 mm). As indicated in Figure 2, the samples were kept at a desired temperature for 20 min to ensure that they were uniformly heated to the same temperature. The power supply was then turned on. An external electrical field was then applied to the sample pair about 1 min before welding began. After a certain welding time, the external electrical field was discharged, the load was removed, and the sample pair was air cooled. This whole process was done in air without any shield gas.

Based on References [5,20,21], a superplastic deformation usually takes place at a deformation temperature greater than 0.5 T_m_ and a strain rate in the range of 10^−4^–10^−2^ s^−1^. Taking this into account, welding parameters for this study were chosen as follows: the temperature was 710–800 °C, the initial superplastic strain rate (${\dot{\varepsilon }}_{0}$) was 1.5 × 10^−4^ s^−1^, the pre-press was 56.6 MPa and the pressure welding time was 0–8 min.

### 2.3. Calculation of Electrical Field Intensity

**Figure 3:**
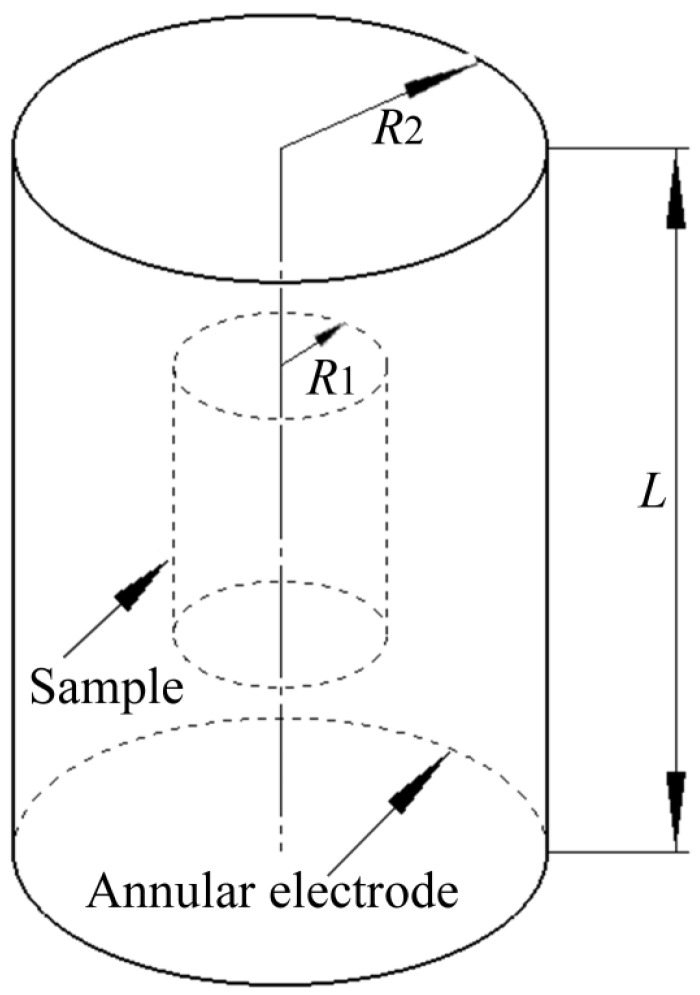
Schematic diagram of the sample and electrode.

As shown in Figure 3, the cylindrical sample acts as one electrode, and another coaxial annular electrode is arranged outside the cylindrical sample. According to Jiang et al. [22], the metal cylindrical sample and the annular electrode can be considered as a coaxial cylindrical capacitor. When *L* ≥ 2*R*_1_, the error caused by the boundary effect is small and can be negligible. The electrical field intensity between the cylindrical sample and the annular electrode can then be expressed as formula (1):

(1) $$\vec{E}=\frac{U}{r\ln\left(R_{2}/R_{1}\right)}\vec{r}$$

Here, *R*_1_ (=7.5 mm) is the radius of the cylindrical sample, *R*_2_ (=13 mm) is the inner radius of the annular electrode, *U* is the potential difference between the annular electrode and the cylindrical sample, and *r* is any distance from the axis of the cylindrical sample (*R*_1_ ≤ *r* ≤*R*_2_). In this study, all the electrical field intensity values are for *r* = *R*_1_, i.e., at the sample surface.

### 2.4. Welding Deformation Measurements and Tensile Tests

**Figure 4:**
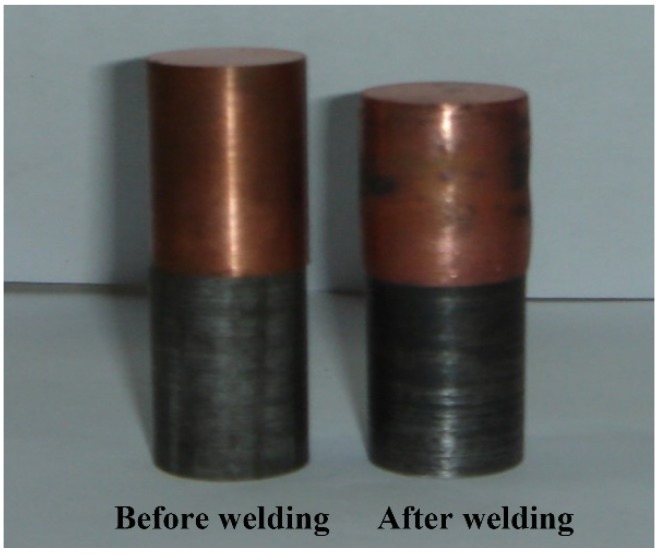
The welding deformation of the samples.

The extent of welding deformation (shown as the Figure 4) was characterized as the expansion strain *ε_d_* = (*d_1_* − *d_0_*)/*d_0_* on both sides of the weld joint, with *d_0_* being the sample diameter before welding and *d_1_* being the maximum diameter after welding. To determine *ε_d_*, both d_0_ and d_1_ were measured with Vernier caliper for all welded samples. For each data point, three measurements were taken to obtain a mean value. The deformation also caused changes of sample heights. Under the welding conditions tested in this paper, the total height reduction was in the range of about 2–5.6 mm, and the height reduction of QCr0.5 was about 3 times greater than that of 40Cr.

After the expansion measurements, welded samples were machined to flat tensile specimens with the weld joint being located in the middle of the specimen. The testing part of the tensile specimen had a dimension of 15 × 5 × 3 mm. All tensile tests were carried out with an AG I-250kN material testing machine (Shimadzu, Kyoto, Japan) at room temperature. Each tensile strength test was repeated 6 times, and then the mathematical average value was determined and used as a data point in the paper. In order to allow a direct comparison of weld strength and strength of base materials, tensile specimens were machined from both base materials and treated with an initial superplastic strain rate of 1.5 × 10^−4^ s^−1^ and pre-press of 56.6 MPa at 740 °C for 4 min before the tensile test. Tensile properties of both base materials are listed in Table 2.

### 2.5. Investigation of Fracture Surfaces and Weld Interfaces

Fracture surfaces and weld interfaces of selected specimens were examined using a JSM-5610LV scanning electron microscope (SEM) (JEOL, Tokyo, Japan). In addition, micro-hardness, as well as Fe and Cu content, were measured across weld interfaces to draw hardness and element profiles.

## 3. Results and Discussion

### 3.1. Material Treatment and Microstructure

**Figure 5:**
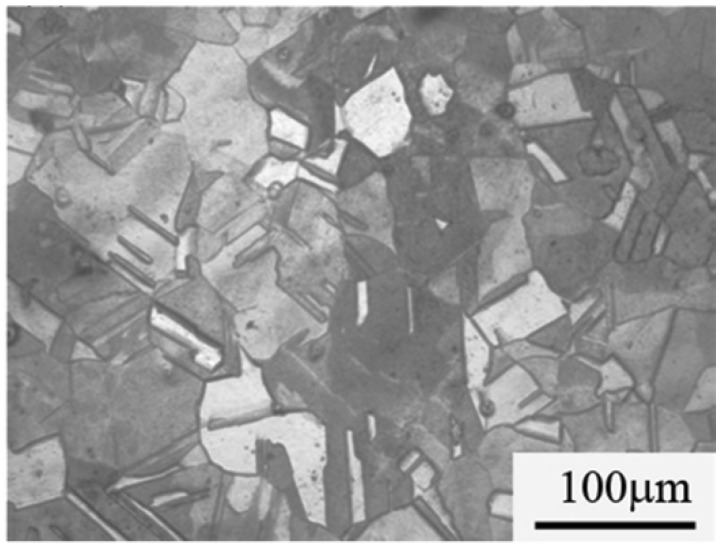
Microstructure of hot-squeezed chrome bronze QCr0.5.

The hot-squeezed chrome bronze QCr0.5 is a quasi-single phase copper alloy. Its microstructure, as shown in Figure 5, consists of the α-matrix with distributed micro-granular Cr. The grain size is about 50 µm. As a quasi-single phase non-ferrous alloy with relatively fine equiaxed grains, the hot-squeezed chrome bronze QCr0.5 meets the microstructure requirement for isothermal super-plasticity and does not need further treatment before welding [23,24].

**Figure 6:**
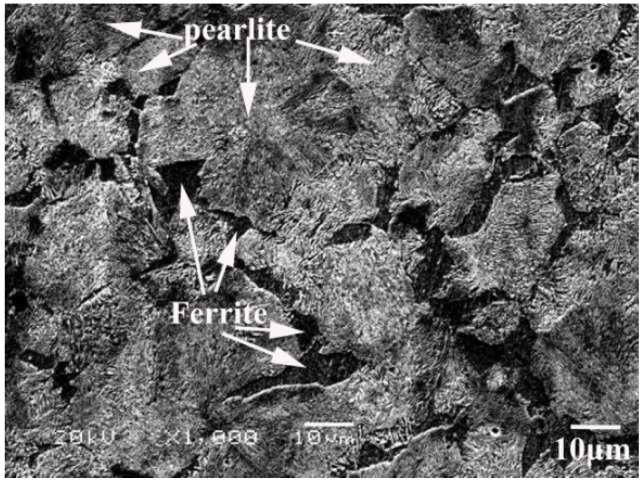
Microstructure of hot-rolled and annealed 40Cr steel.

**Figure 7:**
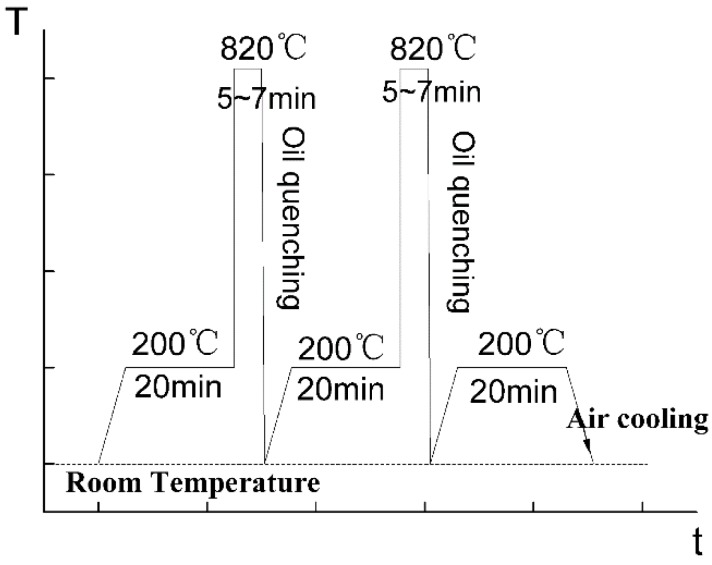
Circulating quenching processing of 40Cr.

**Figure 8:**
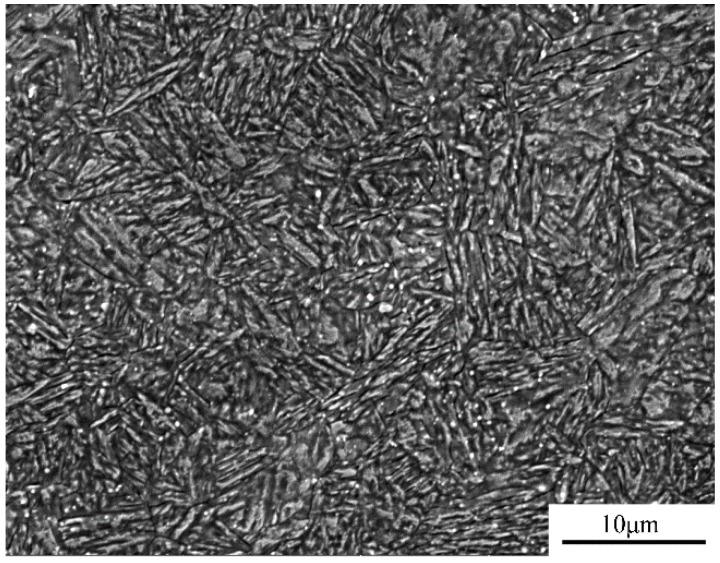
Microstructure of 40Cr steel after ultra-fine treatment.

As shown in Figure 6, the microstructure of hot-rolled and annealed 40Cr steel consists of coarse pearlites (30–50 µm) with small ferrites (5–15 µm). This microstructure is not favorable for a super-plastic deformation during welding [20]. Thus, a so-called ultra-fine treatment is necessary, which adopts a circulating quenching process in the salt bath furnace as depicted in Figure 7. And as shown in Figure 8, the ultra-fine treatment produces a microstructure of acicular martensite that is about 5 µm in length and 0.3 µm in width, which conforms to the microstructure requirement for superplastic welding.

### 3.2. Effect of Electrical Field Intensity

**Figure 9:**
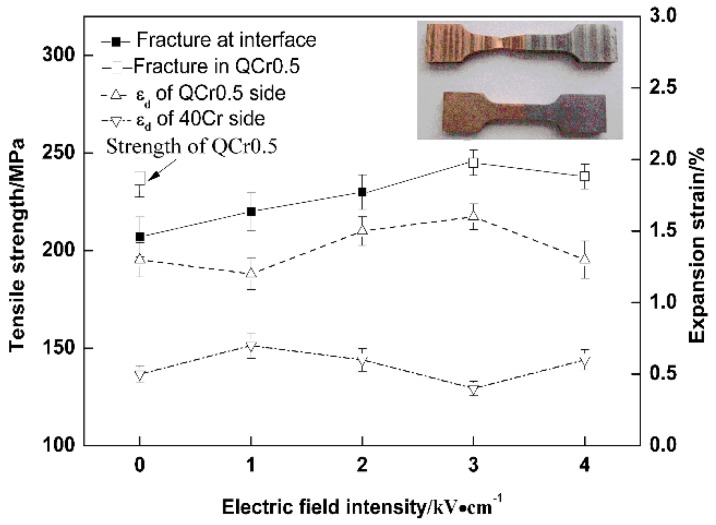
Tensile strength and expansion strain vs. electrical field intensity. (*T* = 740 °C, *t* = 4 min, *P_0_* = 56.6 MPa, ${\dot{\varepsilon }}_{0}$ = 1.5 × 10^−4^ s^−1^).

The tensile strength and expansion strain of weld joints are shown in Figure 9 as functions of electrical field intensity. It can be seen that the tensile strength of weld joints is quite sensitive to external electrical fields. Tensile strength increases alongside with electrical field intensity, until it reaches a peak at 3 kV/cm and decreases a little afterwards. For tensile specimens welded with an electrical field intensity equal to or larger than 3 kV/cm, a fracture occurs in the QCr0.5 base metal (see inserted picture in Figure 9 showing tensile specimens before and after the tensile test), whereas tensile specimens welded with an electrical field intensity of less than 3 kV/cm result in a fracture at weld interfaces. This indicates that the joint strength for a 3 kV/cm and higher electrical field intensity is equal to or exceeds that of the QCr0.5 base metal with the same thermal cycle.

From Figure 9, it is evident that an expansion strain does not show a clear relationship to electrical field intensity. The average expansion strain of the copper alloy QCr0.5 is about 1.5%, 3 times higher than that of 40Cr steel. This can be explained by the softer nature of the copper alloy.

### 3.3. Effect of Welding Temperature

**Figure 10:**
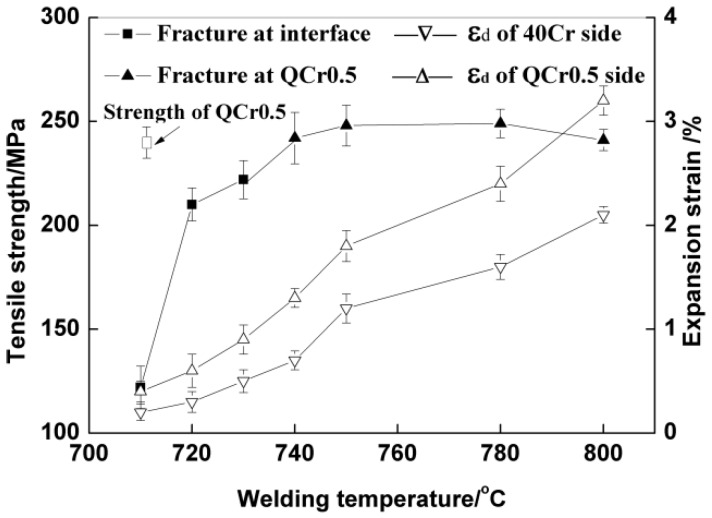
Curves of tensile strength and expansion strain vs. temperature. (*E* = 3 kV/cm, *t* = 4 min, *P_0_* = 56.6 MPa, ${\dot{\varepsilon }}_{0}$ = 1.5 × 10^−4^ s^−1^).

**Figure 11:**
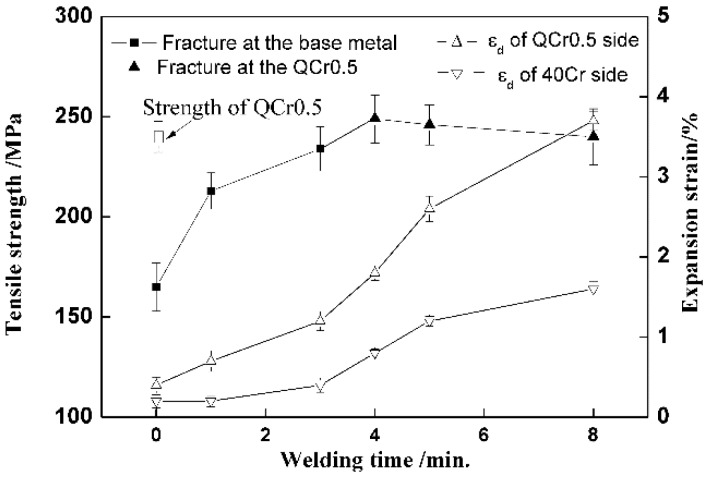
Curves of tensile strength and expansion strain vs. welding time. (*E* = 3 kV/cm, *T* = 740 °C, *P_0_* = 56.6 MPa, ${\dot{\varepsilon }}_{0}$ = 1.5 × 10^−4^ s^−1^).

Since a peak strength is reached at 3 kV/cm in Figure 9, samples shown in Figure 10 and Figure 11 were all welded with 3 kV/cm. In Figure 10, tensile strength and expansion strain are plotted against welding temperature. It can be seen from Figure 10 that with increasing temperature, tensile strength increases rapidly till it reaches its peak at 740 °C and then changes very little in the temperature range of 740–800 °C. When the temperature is below 740 °C, specimens fracture at weld interfaces. In the temperature range of 740–800 °C, fractures occur in the QCr0.5 base metal.

Expansion strain increases with increasing temperature for both QCr0.5 copper alloy and 40Cr steel. Obviously, both materials become much softer with increasing welding temperature.

### 3.4. Effect of Welding Time

Figure 11 shows tensile strength and expansion strain as a function of welding time. As welding time increases, tensile strength increases also until it reaches a peak strength of about 250 MPa at 4 min, when it then decreases slightly. Specimens fracture at weld interfaces for welding times which are less than 4 min, and in the QCr0.5 base metal for 4 min and longer. It is worth noting that a tensile strength of about 165 MPa (about 70% of the peak strength) is measured even when the welding time is for 0 min. This means that even during heating and preheating (see Figure 2), mechanical combination and partial metallurgical bonding can form to some extent, as reported in the literature [3,5].

Expansion strain increases with the extension of welding time for both materials. This can be attributed to a sort of creep effect. The longer the time, the greater the creep deformation.

### 3.5. Observation of Fracture Surfaces and Weld Interfaces

In order to gain a better understanding of how electrical fields influenced weld quality, two selected samples were investigated using SEM: one was welded without an electrical field (*E* = 0 kV/cm), and the other with *E* = 3 kV/cm. The investigations focused on the weld interface and fracture surface of the 40Cr side. Major findings are described below.

**A. Less micro-gaps at interface of sample welded with electrical field**

**Figure 12:**
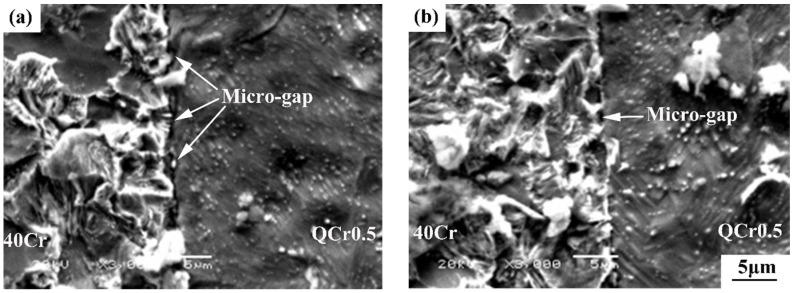
Effect of electrical field intensity on the microstructure of 40Cr/QCr0.5 joints (*T* = 740 °C, *t* = 4 min, *P_0_* = 56.6 MPa, ${\dot{\varepsilon }}_{0}$ = 1.5 × 10^−4^ s^−1^) (**a**) *E* = 0 kV/cm, (**b**) *E* = 3 kV/cm.

Figure 12 shows SEM images of the two welded samples after a common metallographic treatment of grinding, polishing, and etching. Welding defects in the form of micro-gaps can be seen in both samples. However, such defects for the sample welded with *E* = 3 kV/cm (Figure 12b) are clearly less than the sample welded without an electrical field (Figure 12a).

**B. More inter-diffusion in sample welded with electrical field**

**Figure 13:**
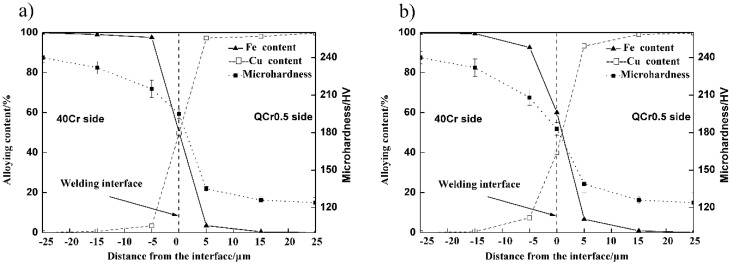
Micro-hardness and composition profiles across the 40Cr/QCr0.5 joint interface. (*T* = 740 °C, *t* = 4 min, *P_0_* = 56.6 MPa, ${\dot{\varepsilon }}_{0}$ = 1.5 × 10^−4^ s^−1^). (**a**) *E* = 0 kV/cm, (**b**) *E* = 3 kV/cm.

Figure 13 shows hardness and element profiles across weld interfaces for both samples welded without (a), and with (b), an electrical field. The shape of the profiles shows clearly that there exists a transition region between copper alloy and steel. Comparing Figure 13a and Figure 13b, one can see that the inter-diffusion is more intense for the sample welded with *E* = 3 kV/cm (Figure 13b) than the sample welded without the electrical field (Figure 13a).

**C. More copper convexes and dimples on fracture surface for the 40Cr sample welded with electrical field**

**Figure 14:**
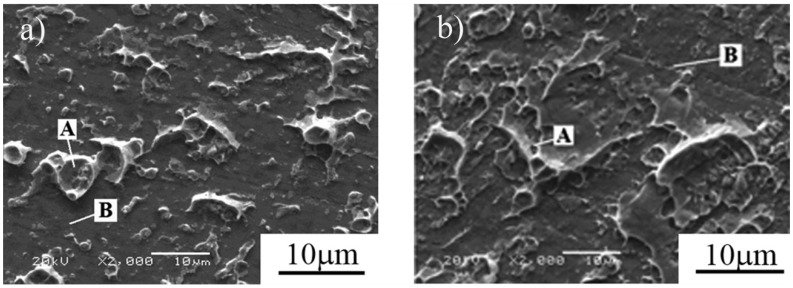
Effect of electrical field intensity on fracture of 40Cr side. (*T* = 740 °C, *t* = 4 min, *P_0_* = 56.6 MPa, ${\dot{\varepsilon }}_{0}$ = 1.5×10^−4^ s^−1^). (**a**) *E* = 0 kV/cm, (**b**) *E* = 3 kV/cm.

In a regular tensile test, the sample welded with *E* = 3 kV/cm fractured in QCr0.5 base metal (see Figure 9). Since we were more interested in observing the fracture surface located as close as possible to the weld interface, a notched tensile specimen was machined from this sample with notches being located precisely on the weld interface, so that the specimen fractured very close to the interface after the tensile test was completed. Figure 14b is the SEM image of the fracture surface of this notched specimen. As a comparison, Figure 14a shows the fracture surface of the sample welded without an electrical field. Both images in Figure 14 were taken with the 40Cr steel side. As a result, two types of regions are visible here: one is covered with a large number of copper convexes and dimples (appearing as light-colored regions and indicated with A in Figure 14), and the other is the original ground area of the 40Cr steel (appearing as gray-colored regions and indicated with B in Figure 14). Comparing the two images in Figure 14, one can see that the fracture surface of the sample welded with *E* = 3 kV/cm shows that a larger area is covered with copper convexes and dimples than the sample welded without an electrical field. This indicates that the weld interface of the sample welded with *E* = 3 kV/cm shows more evidence of metallurgical bonding.

### 3.6. Discussion

In the present work, hot-squeezed chrome bronze QCr0.5 and ultra-fine treated 40Cr steel have been successfully welded using the electro-superplastic solid-state welding technique. Results obtained have shown that the tensile strength of the weld joint can be greatly improved by the application of an external electrical field during the superplastic solid-state welding process. Moreover, the tensile strength of the weld joint can equal or exceed that of the QCr0.5 base metal. This can be attributed to better metallurgical bonding achieved with an electrical field, as confirmed by the above-mentioned 3 major findings in Section 3.5: less micro-gaps, a thicker transition region, and more copper convexes and dimples on the fracture surface for the sample welded with electrical field (Figure 12, Figure 13 and Figure 14).

Micro-gap formation may be understood as being a result of an insufficient vacancy flux during the welding process. At the very beginning of the electro-superplastic solid-state welding process, lots of concentrated vacancies exist at the interface of 40Cr steel and QCr0.5 copper alloy. Vacancies in a metal lattice have a negative charge [6,7,8,9,10,11,12,15,16,17,18], and an electrical field can alter the electrochemical potential of materials [12,13,14,15,16,24]. The charged layer of the metal surface forms in an external electrical field, which can improve vacancy mobility. Therefore, an electrical field can lead to an increased vacancy flux from interior to surface. This way, the vacancy concentration at the welding interface can be reduced, ultimately resulting in less visible micro-gaps at the weld interface in the sample welded with an electrical field (Figure 12).

An electrical field has been found to be able to enhance superplastic deformation of materials [9,13,14,15,17,18]. Larger superplastic deformation means more grain boundary sliding and dislocation movement. Atom diffusion is thus accelerated at the welding interface, and as a result, a thicker transition region is formed in the sample welded with an electrical field (Figure 13).

Due to its softer nature, QCr0.5 starts to plastically deform earlier than the 40Cr during the welding process. This is confirmed by the larger expansion strain measured for the QCr0.5 side (see Figure 9, Figure 10 and Figure 11). Since both vacancy flux and super-plastic deformation can be enhanced by applying an electrical field, a better weld interface of metallurgical bonding is obtained in the sample welded with an electrical field. This, together with the work hardening and alloying effect, makes the weld joint and the QCr0.5 in the vicinity of the weld interface stronger than the QCr0.5 far away from the interface. As a result, samples welded with E ≥ 3 kV/cm resulting in a fracture in the QCr0.5 far away from the interface. On the fracture surface of the notched sample, more copper convexes and dimples were observed (Figure 14), implying that an electrical field is beneficial to obtaining high quality weld joints.

As shown above, a copper alloy QCr0.5 and a steel 40Cr was successfully welded together with an excellent weld strength using the technique reported in this paper. This provides a more economic and time-saving approach to manufacturing different parts involving copper and steel. For example, plungers of plunger pumps are normally made by diffusion welding, which requires a higher welding temperature of 800 °C and a much longer welding time of 1–4 h. Using the technique reported in this paper, this can be done much quicker (4 min) and at a lower temperature (740 °C).

## 4. Conclusions

Hot-squeezed chrome bronze QCr0.5 and ultra-fine treated 40Cr steel have been successfully welded using the electro-superplastic solid-state welding technique. Results obtained have shown that:

1. Tensile strength of the 40Cr/QCr0.5 weld joint can be greatly increased by welding with an external electrical field. This can be attributed to better metallurgical bonding, achieved with an electrical field. Under optimal conditions, the tensile strength can be up to or exceeding that of the QCr0.5 base metal with a tensile fracture occurring in the QCr0.5 base metal. The parameters for the optimal condition include an external electrical field of *E* ≥ 3 kV/cm with no vacuum, no shield gas, a pre-pressure of 56.6 MPa, an initial strain rate of 1.5 × 10^−4^ s^−1^, a pressure welding temperature of 710–800 °C, and a pressure welding time of 0–8 min.
2. The weld interface between 40Cr and QCr0.5 has achieved metallurgical bonding, and there are less micro-gaps, thicker transition regions, and more copper convexes and dimples on the fracture surface of the 40Cr side when applying an external electrical field of *E* = 3 kV/cm.

## Figures and Tables

**Table 1:** Chemical compositions of the two materials.

Mater.	Cu	Cr	Fe	Ni	C	Si	Mn	S	P
QCr0.5	Rest	0.4–1.1	≤0.1	≤0.05	-	-	-	-	-
40Cr	≤0.03	0.8–1.1	Rest	≤0.30	0.37–0.44	0.17–0.37	0.50–0.80	≤0.035	≤0.035

**Table 2:** Mechanical properties and melting points of 40Cr and QCr0.5.

Materials	Hardness	Yield Strength	Tensile Strength	Melting Point
40Cr	240 HV_200g_	510 MPa	708 MPa	≥1538 °C
QCr0.5	124 HV_200g_	140 MPa	238 MPa	1080 °C
